# Ligatures and Sutures—What Material Shall Be Used?1Read at the annual meeting of the American Association of Obstetricians and Gynecologists at Cincinnati, O., September 19, 1889.

**Published:** 1890-01

**Authors:** Clinton Cushing

**Affiliations:** Professor of Gynecology in Cooper Medical College, San Francisco, Cal.; 636 Sutter Street


### LIGATURES AND SUTURES—WHAT MATERIAL SHALL BE USED?[1]

By CLINTON CUSHING, M. D.,

Professor of Gynecology in Cooper Medical College, San Francisco, Cal.

Ligatures and sutures play so important a part in the daily work of the surgeon and gynecologist, that a consideration of the advantages and disadvantages of the various kinds that have been used may prove of service.

Doubtless, nearly all of us finally arrive at a conclusion in favor of some special variety, based upon individual experience and habit. It

1. Read at the annual meeting of the American Association of Obstetricians and Gynecologists at Cincinnati, O., September 19, 1889.

would appear to me that there is as yet no material used for sutures and ligatures that is equally useful and applicable at all times and under all circumstances. If this proposition is true, it is in order to discuss when and where to use certain of the kinds in general use, and the methods of their preparation. The following list embraces all that I am entitled to an opinion upon from personal observation:

Catgut,
Silkworm-gut,
Silk,
Silver wire,
Elastic ligature.

For the past three years, I have prepared all the catgut that I have used with my own hands, and a large experience with it has led me to the conclusion that it is the best obtainable material in certain cases of plastic and abdominal surgery. I purchase from the wholesale dealers in musical instruments several boxes of three sizes of the catgut strings used on the violin. I get the best quality. They are placed in a large open-mouthed bottle filled with sulphuric ether, and allowed to remain for forty-eight hours. When removed, they are very nearly perfectly white, as the ether removes from the catgut all the animal oil. They are then placed in a mixture of three parts alcohol and one part juniper oil, with the addition of three drachms of hydronaphthol to each quart of the fluid. The strings are allowed to remain in this mixture for ten days, when they are ready for use. They are semi-transparent in appearance, are perfectly flexible, and exceedingly strong.

The largest size, or D string of the violin, I use for ligating the pedicle in ovariotomy, and for repairing the perineum. The A string, or middle size, I use for repairing the cervix, and for a buried suture either in the perineum or in the abdominal wall. The E string, or smallest size, I use for ligating adhesions and bleeding vessels in the abdominal cavity.

The D and A strings do not become absorbed by the tissues in less than from seven to nine days, as can be easily demonstrated by observation.

I have also used the A string for stitching together the muscular and peritoneal surfaces of the cervix after hysterectomy for a fibroid tumor. The indications for using catgut as a ligature for the pedicle, after the removal of the tubes or ovaries, will be gone into more fully when speaking of the silk ligature. For the buried suture, in either the perineum or the abdominal wall, the catgut, prepared in this manner, is probably the best. For repairing the cervix, I have found it more satisfactory than any other material, as it does not cut out of the tissues, and holds sufficiently long to secure union. The only disadvantage thus far noticed is that occasionally a small fistulous tract remains along one of the suture holes.

The method of closing the cervix with catgut is exceedingly simple and very rapid. A strong double knot is tied in one end of a piece of gut, about eighteen inches long; the opposite end is frayed out with the back of a knife until perfectly soft and flexible; this is then threaded in a strong needle with a large eye; this triangular-pointed needle is introduced in the usual way at the upper angle of denudation, and drawn until the knot presses against the mucous membrane at the point of introduction of the needle, and then a running or whip-stitch is taken over and over, taking up a goodly amount of the tissue, until the entire line has been closed. I then go back about two stitches, and take two stitches through the already closed part, and then, by making a small running nooze with the last stitch, the catgut is prevented from slipping.

In the perineum, after the denudation in the usual manner, about four sutures are introduced in such a fashion that when they are tied the pelvic floor is lifted upward. The four sutures are then tied with three firm knots, and the ends left an inch or more in length, which prevents the knot from untying or slipping. I use, for this purpose, a very thick and rather short darning-needle, which is the only needle I am able to obtain with a sufficiently large eye to carry the heavy catgut; the suture material in this case, as in the other, being frayed out at the end with the back of a knife, in order to make it soft and pliable. The results in the perineal operations thus far have been all that could be desired in quite a large number of cases.

The advantages of this substance in repairing the perineum are that, in the first place, the material does not tend to cut out, on account of its size; in the next place, it fills the track made by the needle perfectly, because as it becomes softened it swells; in the next place, it apparently does not tend to become septic, as I have not seen any abscess or any noticeable collection of pus about the track of the stitches; and lastly, it does not require the separation of the parts and the worry and anxiety to the patient when the stitches are removed that must take place where silver wire or silkworm-gut, or silk, is used.

Where the perineum and cervix are both repaired at the same sitting, the advantage of catgut for the cervical operation is very decided, because, however well the perineum may unite, it certainly must be considered a detriment to put it upon the stretch with the speculum during the first month, in order to remove the stitches from the cervix, where silver wire, or silk, or silkworm-gut, has been used; and it is true that quite a large proportion of women who require an operation for laceration of the perineum, require also an operation for laceration of the cervix.

The smallest, or E, string is sufficiently strong to ligate safely and successfully almost any bloodvessel in the body, because if a vessel has been thoroughly occluded for one week it may be considered safe, unless the vessel should be badly diseased.

Olshausen, who has written one of the best books on ovariotomy with which I am familiar, says:

> The objections to catgut, that it slips easily, that it is impossible to pull it tightly, and that the knots will loosen, are not justified if the ligature is properly made—that is, with solid, thick, or double threads; if it is drawn firmly, and under vibrating movements of the hands, is knotted three times, the ends in front of the last knot are left long, and a broad mass of tissue allowed to remain.

In another place he says:

> What becomes of the ligature material after an ovariotomy? This depends on circumstances. Carbolized catgut is finally absorbed in the peritoneal cavity, as it is in other living tissues, but it seems to me very probable that the absorption occurs more slowly in the peritoneal cavity than in other tissues. In eleven autopsies in cases of ovariotomy in which the pedicle had been ligated with thick catgut, I found the ligature perfectly firm and unsoftened, although death had not occurred in six cases until the sixth to the thirteenth days.

J. Greig Smith, in his recent work on Abdominal Surgery, says:

> As material for ligature, there is no strong objection to catgut. I have used it and nothing else in more than twenty ovariotomies, and find it perfectly reliable. Its drawbacks are, the trouble necessary for its perfect preparation, and its tendency to deteriorate by keeping; such drawbacks being, in my opinion, of sufficient weight to justify its being displaced by the more handy and equally trustworthy silk twist.

I am convinced that surgeons who have failed with the use of catgut have used it of too small calibre, or have trusted to specimens found in the shops—samples, perhaps, of unknown age.

There are no needles in the instrument shops suitable for the use of heavy catgut, and I have been compelled to use large darning-needles for the perineum, and to have needles especially made for the cervix. These were made from large-size curved needles, broken off an inch and a quarter from the eye, and the broken part ground to a triangular point.

The treatment of the catgut by the ether, by taking out all the animal oil, renders it antiseptic. Were this not true, the ten days' immersion in the alcohol, hydronaphthol, and juniper oil would most certainly do so. The juniper oil renders the gut sufficiently soft and flexible. I believe it to be a mistake to place the animal sutures in corrosive sublimate solutions, as they are thereby rendered more fragile. The catgut should be used direct from the solution of alcohol and juniper oil, and not put into water before using, as the water makes these ligatures soft and slippery, and difficult to handle.

Dr. Henry O. Marcy, of Boston, uses an animal ligature made from the tendinous part of a kangaroo's tail, and claims excellent results therefrom. Fibers from the leg-tendons of the moose have also been used. These sources of supply, however, being limited and uncertain, the probabilities are that our animal ligatures in the future will be the catgut of commerce.

Dr. Nathan Smith, of Connecticut, in 1821, used as ligatures for the pedicle in ovariotomy, strips of leather cut from a kid glove, the pedicle was dropped, the abdomen closed, and the patient recovered; and it has occurred to me that fine strips of buckskin, properly prepared, as I prepare the catgut which I use, might make a most admirable ligature for the pedicle in an ovariotomy, for the reason that it is softer and easier to handle than catgut, does not cut the tissue so much in tying, and will resist absorption longer than catgut, but will probably be absorbed much more quickly than silk. At a future meeting I will give the Association the result of my experiments in this direction.

Silkworm-gut has qualities that are possessed by no other material—that of being absolutely unirritating to the tissues in which it is imbedded, and non-absorbable—at least for a long time. I believe it to be the best material for closing the abdominal wall after an abdominal section. No stitch-hole abscesses have occurred in any of my cases where this material has been used.

It is also an excellent material for perineal sutures. I formerly used it frequently, and with satisfaction. Instead of tying it, and leaving the sharp ends to prick and annoy the patient, I slide over the two ends of the stitch a perforated shot, in which has been tied a loop of strong black silk, an inch in length. When the parts are approximated, the shot is run down to the perineal surface and clamped firmly; then the ends of the stitches are cut off on a level with the surface of the shot. Now, when the sutures are to be removed, the loop of black silk enables you to draw the shot up, so as to make it easy of access, in order that one side of the stitch may be clipped.

The cervix can be united with this same material and in the same manner, and, as the material is not in the least irritating, the suture can be left *in situ* for several weeks; but the operation is not so rapidly and easily done as with catgut.

Silk thread is more generally used by surgeons for sutures and ligatures than any other material, on account of its ease in handling, its non-absorbability, and its strength. These are, undoubtedly, advantages, but it has its disadvantages in being porous, and, therefore, easily absorbing secretions which undergo decomposition and set up local infection. If the silk is first made absolutely aseptic, and the ligature is enclosed in tissue or in a cavity that is also aseptic, no better material could be desired; but these conditions are very different on the surface of the body, or in the mucous canals, or in the peritoneal cavity which has been flooded with pus from a pyosalpinx that has been ruptured during its removal—an accident that occurs frequently in the hands of all operators.

Silk, then, for ligatures in the peritoneal cavity, where no pus or infection exists, is the best; but if the case is one requiring the use of the drainage-tube, catgut should be used. The reasons, it seems to me, are exceedingly simple. If suppuration occurs from any cause, whether imperfect cleansing of the peritoneal cavity, or infection introduced through the drainage-tube, and the ligature material of silk becomes involved, the probabilities are that a fistula will remain until the ligature comes away, either forming the nidus for an abscess which opens into the rectum or the bladder, or discharges through the opening left at the site of the drainage-tube. The ligature ultimately is thrown off, and the case recovers.

I doubt not, there are few who have had much to do with abdominal surgery, especially in the removal of the Fallopian tubes in cases of pyosalpinx, but have had this unfortunate experience of a fistula being left after the operation; and it is probable that this fistula was due to the existence of an infected ligature in a considerable proportion of cases. Twice during the past year, I have been forced to enlarge the opening left by the drainage-tube and remove the ligature from the pedicle, in order to secure the healing of fistulæ.

In a recent discussion before the Obstetrical Society of Philadelphia, Dr. Howard A. Kelly says:

> I have known cases of infection of ligatures from a tube left in for a protracted length of time. I have frequently had ligatures sent to me by patients. In all these cases the fistulæ, which have persisted for weeks or months, have healed when the ligature has been rejected.

Dr. Goodell says:

> I have on several occasions fished ligatures out of a fistulous tract, and thus cured it. I am satisfied that one patient, on whom I performed ovariotomy a year ago, and who is suffering from a fistula, has a ligature at the bottom of the tract.

Several other members of the Society also cited one or more cases of fistula following abdominal operations, that failed to heal until the loop of silk used in tying the pedicle had come away.

The argument, it seems to me, with our present light, is easy of solution. In a simple case of ovariotomy where no pus exists, or there is no need for a drainage-tube, silk, properly prepared, is the best material. If, on the other hand, it is necessary to leave a drainage-tube in the cavity, or the cavity of the peritoneum has been infected with pus, catgut should be used, for the simple reason that it will disappear by absorption in ten days, and will not thus be a cause of fistula or abscess.

There is a strong feeling in the minds of nearly all, that silk is the safest material for ligature in ovariotomy, but I doubt not that properly prepared catgut, of the calibre of the D string of the violin, is strong enough and safe enough for any pedicle, however large.

I have had made at the silk factory at San Francisco a kind of heavy silk for the pedicle in ovariotomy cases, which seems to me to possess some advantages over any that I have seen. It is made of the best quality of Chinese silk, thoroughly washed, and is extremely soft; it is very loosely twisted, and when tied applies itself smoothly to the parts without cutting; the knot does not slip, as is the case with the harsh and tightly twisted silk found in the shops. The variety that I saw used in Great Britain by Keith, Tait, Bantock, and Thornton, is made of unwashed silk, with the natural gum still in it. It is harsh, twisted very tightly, and, under the finger, feels like coarse linen. This, when tied, acts almost like a piece of wire, and the knot tends to slip much more than it does with the soft and loosely twisted thread.

I herewith present to the Association a sample of the silk made here. The silk used for ligating vessels is the kind sold in the dry-goods shops on spools, and marked "double E." It is very strong, and much cheaper than that bought in small hanks at the instrument shops.

In injuries of the bowel, and in a resection of the bowel for stricture, I have always used, for uniting the cut portions of the gut, fine sewing silk, and I think it is generally agreed that no material is so satisfactory as this for repairing a wound of the intestine.

I prepare the silk by first winding it loosely on a wooden reel. This is then boiled in a saturated solution of water and hydronaphtho for half an hour. It is then placed in absolute alcohol, containing hydronaphthol, for twenty-four hours. It is then wound upon spools and is ready for use, the spool being dropped into a saturated solution of hydronaphthol and water each time before using.

Silver wire I use less each year. For some years I have used it only in cases of vesico-vaginal fistula, but I doubt not silkworm-gut would be equally good material in such cases. Its advantages of being unirritating to the tissues and easily made aseptic, are qualities which hold equally good of the silkworm-gut. Its disadvantages are, that its introduction is attended with more difficulty than the sutures that have been already mentioned, on account of its stiffness, as we must first introduce a loop of silk and afterward draw the wire into position; whereas, with the silkworm-gut it can be threaded directly into the eye of the needle and placed *in situ* more easily and more rapidly. Furthermore, the silkworm-gut is less costly. It has seemed to me, too, that silver wire tends to cut out of the tissues more readily than the silkworm-gut.

The elastic ligature I have only used to surround the stump in amputation of the body of the uterus for fibroid tumor. Where the stump is treated extra-peritoneally, the elastic ligature has seemed to me to have many advantages over other methods of controlling hemorrhage; but my success of late in the treatment of the stump according to the plan of Schroeder, by ligating the principal vessels at the side of the cervix, excavating the surface of the stump, and suturing first the muscular and afterward the peritoneal coats together with heavy catgut, and closing the abdomen, have been such that I have seldom used the elastic ligature. Olshausen has applied it to the pedicle in ovariotomy in a large number of cases, and, he claims, with good success; and he has demonstrated by subsequent examinations that the rubber ligature becomes encysted and does no harm. Of this, however, I know nothing personally.

636 Sutter Street.

